# BRM270 Inhibits the Proliferation of CD44 Positive Pancreatic Ductal Adenocarcinoma Cells via Downregulation of Sonic Hedgehog Signaling

**DOI:** 10.1155/2019/8620469

**Published:** 2019-02-24

**Authors:** Do Luong Huynh, Hyebin Koh, Nisansala Chandimali, Jiao Jiao Zhang, Nameun Kim, Tae Yoon Kang, Mrinmoy Ghosh, Meeta Gera, Yang-Ho Park, Taeho Kwon, Dong Kee Jeong

**Affiliations:** ^1^Laboratory of Animal Genetic Engineering and Stem Cell Biology, Advanced Convergence Technology & Science, Jeju National University, Jeju 63243, Republic of Korea; ^2^Chongqing Key Laboratory of Forage and Herbivore, College of Animal Science and Technology, Southwest University, Chongqing 400715, China; ^3^Jeju Special Self-Governing Province Livestock Promotion Agency, 13 Chuksanmaeul-gil, Jeju 63078, Republic of Korea; ^4^Department of Biotechnology, Division of Research and Development, Lovely Professional University, Punjab 144411, India; ^5^BRM Institute, Seoul 01756, Republic of Korea; ^6^Primate Resource Center, Korea Research Institute of Bioscience and Biotechnology (KRIBB), Jeongeup-si, Jeonbuk 56216, Republic of Korea

## Abstract

Pancreatic cancer has a poor survival rate as compared to other types of cancer. Surface marker CD44 plays important role in epithelial-mesenchymal transition and cancer stem cell phenotype. Therefore, targeting CD44 positive pancreatic cancer cells might enhance therapies effectiveness. Our previous studies indicated the antitumorigenesis effect of BRM270 in osteosarcoma, lung cancer, and glioblastoma; however there is no evidence on BRM270 impacts on pancreatic cancer growth. In this study, we investigated the effect of BRM270 on the isolated CD44 positive pancreatic ductal adenocarcinoma cells (CD44^+^ PDAC). Results showed that CD44 positive cells undergo apoptosis induced by BRM270. Moreover, BRM270 also inhibits stemness and metastasis traits in CD44^+^ PDAC via Sonic hedgehog signaling pathway and SALL4 expression.* In vivo* study indicated that tumor growth derived from CD44^+^ PDAC was suppressed as daily uptake by BRM270 5 mg/kg. These data suggest the alternative approach in antipancreatic tumorigenesis via herbal plants extract and selectively targeting CD44^+^ PDAC cells in tumor.

## 1. Introduction

Survival rate in pancreatic cancer is extremely lower as compared to other cancers [1, 2]. Pancreatic ductal adenocarcinoma (PDAC) accounts for 80% of pancreatic cancer and becomes one of the most death cases in the world [3, 4]. Many attempts to cure PDAC have been deployed. However, therapeutic efficiency remains low, due to its silent symptoms, lack of early diagnosis, or effective therapies [3, 4]. Many evidences indicated that CD44 expression is strongly associated with epithelial-mesenchymal transition (EMT) and cancer stem cell (CSC) phenotypes such as tumor invasion, metastasis, recurrence, or chemoresistance [5]. CD44 is also considered as one of the CSC markers in various types of tumors [5, 6]. Furthermore, clinical reports showed that high expression of CD44 is linked to poor survival rate [6]. Therefore, new findings in anticancer cells targeting high CD44 expression could be a promising approach [6, 7].

Sonic hedgehog (Shh) signaling pathway plays important role in tumorigenesis, including tumor initiation, promotion or metastases in skin, leukemia, lung, brain, and gastrointestinal cancers [8]. In pancreatic cancer, evidences also indicated that Shh signaling pathway regulates tightly pancreatic CSCs stemness and metastatic traits [9, 10]. Many target genes such as transcription factors of pluripotency (Oct-4, Sox-2, Nanog, and c-Myc) or epithelial-mesenchymal transition (EMT) genes (MMP-9, CXCR4, Snail-1, and N-cad) are upregulated via Shh stimulation, resulting in high metastatic phenotype and drug resistance or tumor relapse as well [11, 12]. Therefore, the inhibition of Shh signaling becomes the main concern in antipancreatic cancer.

BRM270 in previous studies shows its effects in antitumorigenesis [13–15]. As a natural extract, BRM270 inhibits the proliferation of lung adenocarcinoma and glioblastoma stem cells* in vitro *and* in vivo*. In this study we aim to examine the effects of BRM270 on CD44 positive (CD44^+^) PDAC cells. BRM270 significantly represses the proliferation and metastasis and deactivates SALL4-mediated CSCs maintenance and Shh expression and activities, thus favoring antipancreatic tumor treatment. Taken together, BRM270 might be a multitarget arrow in cancer treatment, targeting both in CSCs maintenance and metastasis phenotype.

## 2. Materials and Methods

### 2.1. Reagents

BRM270 supplied by BRM Institute (Seoul, Korea) was extracted by methanol/ethanol, followed by rotary concentration. Pellet was dissolved in DMSO (Sigma-Aldrich, MO, USA) and stored at −20°C for further analysis. Polyclonal antibodies against Bcl-2, Bcl-xL, Caspase-3, CD133, CD44, c-Myc, CXCR4, p53, GADPH, Gli-1, Nanog, N-cadherin, Oct-4, SALL-4, Shh, Snail-1, Sox-2 antibodies, and horseradish peroxidase- (HRP-) conjugated anti-rabbit or anti-mouse IgG were purchased from Santa Cruz Biotechnology (Santa Cruz, CA, USA).

### 2.2. Isolation of CD44 Positive PDAC Cells

Cells in log phase were used for magnetic-activation cell sorting (MACS) separation with CD44 microbeads (Miltenyi Biotec, Germany) as manufacturer's protocol. Subpopulations of CD44 negative (CD44^−^) and CD44 positive (CD44^+^) were subjected to further analysis.

### 2.3. Cell Culture

PANC-1 and BxPC-3 cells were from cultured DMEM (Gibco, CA, US), supplemented with 10% FBS (Welgene, Korea) and 1% antimycotic type and maintained in a humidified atmosphere of 5% CO_2_ in incubator at 37°C, two passages weekly. CSCs after isolation by magnetic activated cells sorting (MACS, Miltenyi Biotec, Germany) were maintained in DMEM/F12 plus with 2% B27, 10 ng/mL hEGF, (Sigma-Aldrich, MO, US), and 10 ng/mL bFGF (KOMA Biotech, Seoul, Korea) [16].

### 2.4. Flow Cytometry Assay

Cells after isolation by MACS were certified by CD44-APC (Miltenyi Biotec, Germany) FACS analysis as manufacturer's protocol. For apoptosis assay, in brief cells after 48 h treatment by BRM270 were resuspended in 100 *μ*l binding buffer containing 5 *μ*l Annexin V-FITC conjugated antibody and 5 *μ*l propidium iodide for exactly 10 min in the dark at room temperature. Cells were then analyzed on BD Accuri C6 cytometer (BD Biosciences, NJ, US).

### 2.5. Cell Viability

5 × 10^3^ cells were seeded into a 96-well plate and incubated 24 h before treatment with or without indicated BRM270 concentrations. After 48 h, cell cytotoxic effects were measured by EZ-Cytox kit (Daeil Lab, Seoul, Korea) according to the manufacturer's protocol. The cell viability results are presented as the ratio of optical density at 450 nm (OD_450_) that was calculated using the following formula: (%) cell viability = (OD treatment groups or control groups/OD vehicle control group) × 100%.

### 2.6. Immunocytochemistry Staining

Cells were fixed by 3.7% paraformaldehyde (PFA). Before overnight staining with primary antibodies of interest, cells were blocked by phosphate-buffered saline 0.1% Tween-20 (PBST 1X) with 3% BSA. After washing 2 times with PBST 1X, secondary antibodies were added and followed by 2 h incubation. After two washes by PBST 1X, cells were stained 10 min with 4′,6-diamidino-2-phenylindole (DAPI) before capturing on microscope.

### 2.7. Western Blotting

Protein quantity in lysates was determined using BCA assay. Proteins were separated on a 12% SDS-PAGE, transferred electrophoretically (Bio-Rad, CA, USA) onto a polyvinylidene fluoride (PVDF) membrane, and blocked with 5% nonfat milk powder (w/v) in PBST 1X for 1 h at room temperature, followed by incubating with primary antibodies or with anti-GADPH mouse monoclonal antibody as an internal control overnight at 4°C and with appropriate HRP-conjugated secondary antibodies at RT for 2 h. The bands were captured by ImageQuant™ LAS 4000 mini Fujifilm [16].

### 2.8. Clonogenic Assay

1 × 10^3^ cells were seeded in 6-well plate at 37°C/ 5% CO_2_. After 7 days for incubation, cells were fixed with 3.7% formaldehyde and stained with 0.05% crystal violet/washing by PBS 1X before capturing [16].

### 2.9. Sphere Formation

1 × 10^3^ cells were seeded into ultralow attachment 6-well plate (Corning, NY, US) in DMEM/F12 plus with 2% B27, 10 ng/mL hEGF (Sigma-Aldrich, MO, US), and 10 ng/mL bFGF (KOMA biotech, Seoul, Korea) with and without BRM270 treatment. After 7 days of incubation, spheres were captured by microscope.

### 2.10. In Vitro Cell Migration and Invasion Assays

Cell migration assay was performed using 8-*μ*m pore size hanging cell-inserts (Merck Millipore, MA, US). 1 × 10^5^ cells in 0.5% FBS-DMEM were seeded in upper chamber while lower chamber was filled with 20% FBS. After 48 h incubation, migrating cells were stained with 0.05% crystal violet (w/v). The number of migrated cells on the lower surface of the membrane was counted under a microscope in five random fields at 100 ×. For cell invasion assay, all procedures were carried out as in the migration assay, except that Matrigel matrix growth factor reduced basement (BD Biosciences, NJ, US) (3.5 mg/mL) was coated on the upper chamber according to the manufacturer's protocol.

### 2.11. Wound Healing Assay

Cells were used in wound healing assay as per manufacturer's protocol. Briefly, cells at log phase were seeded into IncuCyte ImageLock 96-well microplates, reaching 98%–100% confluence after overnight. Monolayer of cells was scratched by wound-maker and underwent real-time imaging by IncuCyte system (Essen Bioscience, MI, US) as indicated time points.

### 2.12. In Vivo Evaluation

Mice were maintained according to a protocol approved by the Institutional Animal Care and Use Committee of Jeju National University (Jeju, Korea). Tumors were induced by subcutaneously injecting 1 × 10^6^ cells in 100 *μ*l mixed volume Matrigel (Sigma-Aldrich, MO, USA) and PBS into the flanks of 6-week-old nude male BALB/c-nu mice (*n*=3/each group). Tumors were measured every 7 days by using caliper. BRM270 was orally supplied every day at dose of 5 mg/kg. The tumor volume (V = W × L × H/2) was evaluated by length (L), height (H), and width (W). Mice were sacrificed at day 35 after cell injection.

### 2.13. Statistical Analysis

Statistical analysis was performed using Graphpad Prism 6.02. Data are expressed as mean ± standard deviation (SD). Experimental differences were examined using ANOVA and Student's* t*-tests, as appropriate.* P *values of <0.05 were considered to indicate statistical significance.

## 3. Results

### 3.1. Isolation of CD44 Positive PDAC Cells

**Figure 1:**
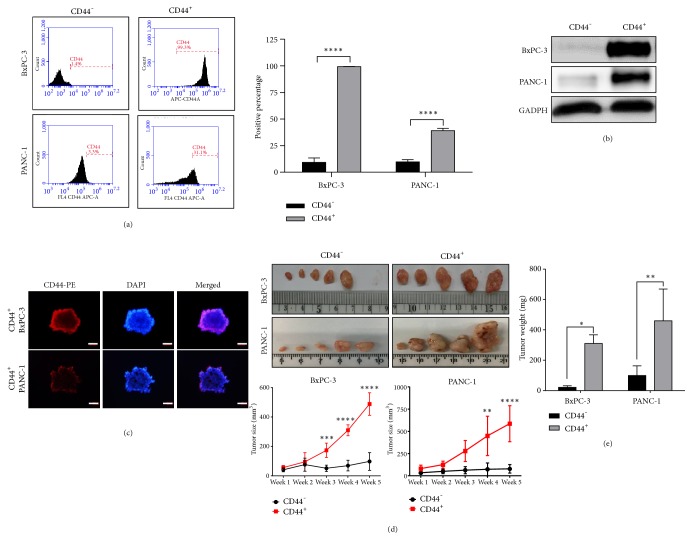
Isolation of CD44^+^ PDAC cells. (a) FACS analysis of CD44 surface marker in positive and negative CD44 BxPC-3 and PANC-1 after MACS separation. (b) Western blotting of lysates after MACS separation. (b) Immunocytochemistry staining of CD44, scale bar 20 *μ*m. (d) Comparison of CD44^+^ and CD44^−^ induced tumor sizes. (e) Tumor weights of CD44^+^ and CD44^−^ PDAC cells. Significant differences of values are compared to values of negative and positive CD44 cells and marked as follows: *∗P< 0.05; ∗ ***∗***P< 0.01; ∗∗∗P< 0.001; ∗∗∗∗P< 0.0001.*

It is implied that CD44 surface marker is associated with PDAC malignance [6]. In this study, BxPC-3 and PANC-1 were chosen for MACS separation of the CD44^+^ cells. Results showed that proportion of CD44 was differed significantly after separation, represented by FACS analysis and Western blotting (Figures 1(a) and 1(b)). Immunocytochemistry staining showed the CD44 expression in tumor spheres of CD44^+^ BxPC-3 and PANC-1 (Figure 1(c)). Regarding tumor formation, cells with high expression of CD44 showed the dominance in tumor sizes and weights (Figures 1(d) and 1(e)). These data suggested that CD44^+^ PDAC cells are more malignant as compared to CD44 negative subpopulation.

### 3.2. BRM270 Inhibits the In Vitro Malignance of CD44 Positive PDAC Cells

**Figure 2:**
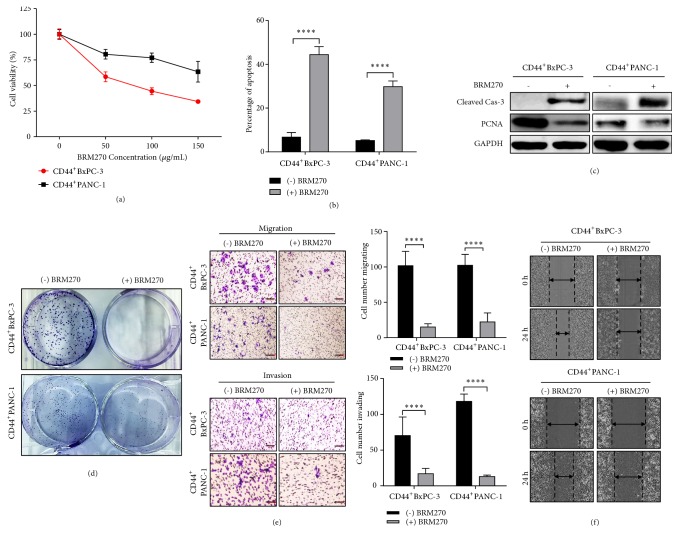
BRM270 suppresses the malignance of CD44^+^ PDAC cells. (a) Dose-dependent inhibitory effects of BRM270 to CD44^+^ PDAC cells. (b) The apoptosis of CD44^+^ PDAC cells exposed by BRM270, detected by FACS Annexin V. (c) Western blotting of apoptotic proteins in lysates with and without 50 *μ*g/mL BRM270 treatment. (d) Clonogenic assay of CD44^+^ PDAC cells with and without 50 *μ*g/mL BRM270 treatment. (e) Migration and invasion assay of CD44^+^ PDAC cells with and without 50 *μ*g/mL BRM270 treatment, scale bar 100 *μ*m. (f) Wound healing assay of CD44^+^ PDAC cells with and without 50 *μ*g/mL BRM270 treatment. Significant differences of values are compared to values of positive CD44 cells with and without BRM270 treatment and marked as follows: *∗∗∗∗P< 0.0001.*

Cells with high expression of CD44 were exposed to BRM270 50 *μ*g/mL. After 48 h treatment, signs of apoptosis were examined by cell viability assay, FACS Annexin V, and Western blotting. Results showed that BRM270 inhibited the proliferation of CD44^+^ PDAC cells dose dependently (Figure 2(a)). FACS analysis showed the increases of apoptotic cells under BRM270 treatment (Figure 2(b)). Furthermore, there were the activation of caspase-3 and downregulation of PCNA (Figure 2(c)). BRM270 also decreased the clonogenicity of CD44^+^ PDAC cells (Figure 2(d)). These data indicated that BRM270 induced the apoptosis of CD44^+^ PDAC cells. Moreover, inhibitory effects of BRM270 to CD44^+^ PDAC malignances also were noted via cell mobility assay. BRM270 treatment decreased considerably the numbers of cells migrating and invading (Figure 2(e)). Furthermore, BRM270 inhibited notably the wound closures of CD44^+^ PDAC after 24 h treatment (Figure 2(f)). Taken together, these data suggested that BRM270 prevents effectively the* in vitro* malignances of CD44^+^ PDAC cells.

### 3.3. BRM270 Represses Self-Renewal Capacity of CD44 Positive PDAC Cells

**Figure 3:**
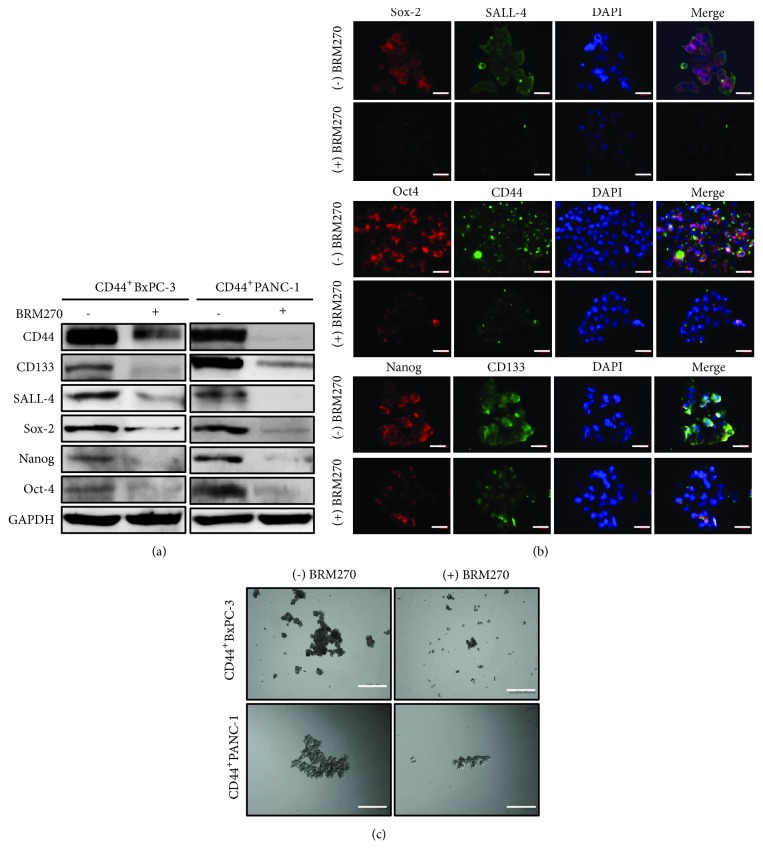
BRM270 inhibits self-renewal capacity of CD44^+^ PDAC cells. (a) Western blotting of stemness genes in lysates treated with and without 50 *μ*g/mL BRM270. (b) Immunocytochemistry staining of stemness genes in CD44^+^ PDAC cells with and without 50 *μ*g/mL BRM270 treatment, scale bar 20 *μ*m. (c) Sphere formation assay of CD44^+^ PDAC cells with and without 50 *μ*g/mL BRM270 treatment, scale bar 100 *μ*m.

Self-renewal capacity is driven by various stemness genes such as CD133, SALL4, Oct4, Sox-2, and Nanog [17, 18]. To elucidate the inhibitory effects of BRM270 on CD44^+^ PDAC self-renewal capacity, cells were proceeded to Western blotting, immunocytochemistry staining, and tumor sphere formation. Results showed that there were downregulations of stemness genes, including CD44, CD133, SALL4, Oct4, Sox-2, and Nanog under 50 *μ*g/mL BRM270 treatment (Figure 3(a)). Levels of these genes were repressed in immunocytochemistry staining (Figure 3(b)). Furthermore, tumor sphere formations of CD44^+^ PDAC cells were inhibited in the presence of 50 *μ*g/mL BRM270 (Figure 3(c)). These data suggested that BRM270 inhibits efficiently the self-renewal capacity of CD44^+^ PDAC cells via downregulation of stem cell factors.

### 3.4. BRM270 Restrains CD44^+^ PDAC Derived Tumor Growth via Sonic Hedgehog Signaling

**Figure 4:**
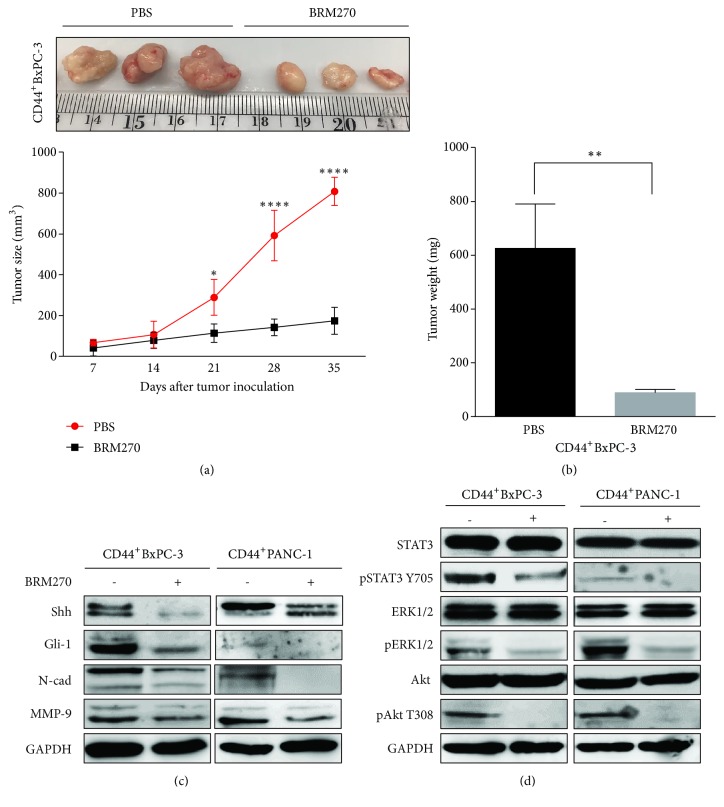
BRM270 restrains CD44^+^ PDAC cells derived tumor growth via downregulating Sonic hedgehog signaling pathway. (a) Sizes of tumors with and without 5 mg/kg BRM270 treatment after 35-day inoculation. (b) Weights of tumors with and without 5 mg/kg BRM270 treatment. (c) Western blotting of epithelial-mesenchymal transition (EMT) genes in lysates treated with and without BRM270 50 *μ*g/mL. (d) Western blotting of signaling pathways in lysates treated with and without BRM270 50 *μ*g/mL. Significant differences of values are compared to values of groups treated and nontreated with BRM270 and marked as follows: *∗P< 0.05; ∗∗P< 0.01; ∗∗∗∗P< 0.0001.*

Our previous studies showed antitumor effects of BRM270 in lung cancer and glioblastoma while there are no changes of body weight loss or side effects [13–15]. In this study, we examined whether BRM270 can suppress tumorigenesis derived CD44^+^ PDAC cells. After 5 weeks of inoculation, tumors treated with 5 mg/kg BRM270 were significantly reduced as compared to PBS-treatment group (Figures 4(a) and 4(b)). Regarding signaling pathway, BRM270 downregulated Shh/Gli1 expressions, leading to the suppression of N-cad and MMP9 expression (Figure 4(c)). Moreover, there was dephosphorylation of STAT3, ERK1/2, and Akt downstream signaling pathways after exposure to 50 *μ*g/mL BRM270 (Figure 4(d)). These data suggested that BRM270 suppresses the CD44^+^ PDAC cells derived tumor growth via Sonic hedgehog signaling pathway.

## 4. Discussion

Pancreatic cancer often has poor prognosis, and PDAC accounts for 85% in most pancreatic cancer diagnosed cases [19]. CD44 existence implies the EMT in pancreatic cancer, cancer stem cells, or drug resistance [6]. Therapies targeting CD44 show their efficiencies in prevention of cancer [6, 7, 20]. Our study indicated that BRM270 presents strong inhibitory effect on CD44^+^ PDAC cells via induction of cancer cell death, inhibitions of migration, invasion, and wound healing behaviors. Furthermore, BRM270 exhibits its potential in antistemness factor expressions such as CD133, Sox-2, Nanog, or Oct4. BRM270 also inhibits SALL-4, which acts as an oncofetal stemness gene and governs the expressions of stem cell factors and CD44 [21], resulting in CD44 diminution. SALL-4 existence in PDAC cells is believed to facilitate metastatic characteristics, via reactive oxygen species (ROS) regulation [16]. Therefore, suppression of SALL-4 by BRM270 would possibly decrease metastasized death cases.

Chemoresistance and early metastatic spread induced by pancreatic CSCs are severe, resulting in the failure of current treatments [22]. Any therapies conquering chemoresistance and metastasis would efficiently advance antipancreatic cancer. Shh signaling is activated highly and involves notably such characteristics [10]. Inhibition of Shh signaling pathway restores chemosensitivity and frustrates self-renewal capacity in pancreatic CSCs [11, 12]. In this study, BRM270 significantly suppresses Shh/Gli1 signaling pathway, leading to the downregulations of EMT genes, and consequently inhibits metastatic phenotype in CD44^+^ PDAC cells. Therefore, BRM270 could be the new adjuvant, partly sensitizing pancreatic CSCs to chemo-drugs while directly hitting self-renewal capacity or metastatic spread.

At present, anti-PDAC by gemcitabine treatment recently shows resistance [8]. Furthermore, gemcitabine is believed to promote immunosuppressive tumor environment or invasiveness in pancreatic cancer [23, 24]. Therefore, minimizing gemcitabine dose by combination with other synergizing drugs or finding new replacement of chemotherapies is urgent. In this study, we selected two cell lines, the most sensitive and resistant to gemcitabine BxPC-3 and PANC-1, corresponding to wild type and mutant K-RAS, to investigate BRM270 effects. Results show that K-RAS with wild type (BxPC-3) responds sensitively to BRM270 as compared to the mutant (PANC-1). This event is similar to gemcitabine responses from these cell lines [25]. Possibility is that K-RAS mutant might subconsciously activate downstream signaling pathways including Akt or ERK, eventually resulting in cell survival [26].

Another possibility might be that the sensitivity to gemcitabine or BRM270 related to p53 status. BxPC-3 and PANC-1 have mutant* TP53* at Y220C and R273H, respectively. Y220C is believed to destabilize p53 while R273H affects p53 binding function [27]. BRM270 might help stabilize p53 conformation, resulting in more wild type p53, and consequently drives apoptosis once treatment occurs. Therefore, BxPC-3 with Y220C* TP53* is sensitive to BRM270. Generally, BRM270 in our study inhibits both K-RAS mutant and wild type or* TP53* mutant activities by suppressing Akt and ERK1/2 or STAT3 phosphorylation signaling, presenting its extensive inhibitory effects on the main mutations causing PDAC. Definitely, there are many dark sides needed to be uncovered, such as the effects of BRM270 on tumor environment at early metastasis stage or its behaviors in anti-metastasizing or anti-circulating pancreatic tumor cells or the combination between BRM270 and gemcitabine in pancreatic cancer intervention. Nevertheless, these findings initiatively assert BRM270 uses in attempts of antipancreatic cancer, the first step for safe therapies.

## Data Availability

The data used to support the findings of this study are available from the corresponding author upon request.
